# Exploring Hemodynamic Responses Using Mirror Visual Feedback With Electromyogram-Triggered Stimulation and Functional Near-Infrared Spectroscopy

**DOI:** 10.3389/fnhum.2019.00060

**Published:** 2019-02-26

**Authors:** Yuji Inagaki, Kazunori Seki, Hitoshi Makino, Yuichirou Matsuo, Tamaki Miyamoto, Katsunori Ikoma

**Affiliations:** ^1^Department of Rehabilitation Medicine, Graduate School of Medicine, Hokkaido University, Sapporo, Japan; ^2^Sendai Clover Clinic, Sendai, Japan; ^3^Department of Physical Therapy, Hokkaido Bunkyo University, Eniwa, Japan; ^4^Hokkaido Medical Center, Sapporo, Japan; ^5^Department of Psychiatry, Graduate School of Medicine, Hokkaido University, Sapporo, Japan

**Keywords:** near-infrared spectroscopy, postcentral gyrus, electric stimulation, mirror visual feedback, electromyogram

## Abstract

In recent years, mirror visual feedback (MVF) therapy combined with electrical stimulation (ES) have been proposed for patients with hemiparesis. However, the neurophysiological effect remains unknown. We investigated the effects of MVF by itself and along with electromyogram-triggered ES (ETES) on hemodynamic responses using functional near-infrared spectroscopy (NIRS). Eighteen healthy subjects participated in this study. We measured changes in brain oxygenation using 48 NIRS channels. We investigated the effects of three main factors of visual feedback (observation of a mark, right hand, and hand movements *via* mirror) with or without ES on bilateral precentral gyrus (PrG), postcentral gyrus (PoG), supplementary motor area (SMA), supramarginal gyrus area (SMG), and angular gyrus (AG) to determine the contribution of each factor. The results showed that the left PoG was significantly more activated when performing mirrored tasks (MT) than when performing circle or Right-hand Tasks (RTs). In addition, the right PoG and right SMA in MT were significantly more activated than in MT + ES cases. Our findings suggested that observation of movements through the mirror caused activation of the postcentral gyrus rather than the PrG, and MVF along with ETES decreased cortical activation.

## Introduction

In mirror visual feedback (MVF) therapy, subjects perform movements of the unimpaired limb while watching a mirrored reflection of their movements superimposed over the unseen impaired limb; this technique was introduced by Ramachandran and Rogers-Ramachandran (1996). MVF therapy was initially developed to treat phantom pain; however, substantial evidence has demonstrated the efficacy of MVF therapy in motor or sensory recovery in patients with stroke (Altschuler et al., 1999; Sathian et al., 2000; Yavuzer et al., 2008) and complex regional pain syndrome (McCabe et al., 2003; Moseley, 2004).

The neurophysiological effects induced by MVF have been investigated using various methods including transcranial magnetic stimulation (TMS), electroencephalography (EEG), magnetoencephalography (MEG), and functional magnetic resonance imaging (fMRI). TMS studies have shown that MVF increases human primary motor cortex (M1) activity of the stationary hand in healthy subjects (Garry et al., 2005; Fukumura et al., 2007; Kumru et al., 2016). In addition, MEG and EEG studies have shown that M1 activation is increased by MVF in healthy subjects (Tominaga et al., 2011; Debnath and Franz, 2016). Likewise, fMRI studies have revealed significant activation of the sensorimotor cortex, cerebellum, and visual areas ipsilateral to the moving hand (Wang et al., 2013; Milde et al., 2015). A resting state fMRI study (Rjosk et al., 2017) revealed functional alterations in the bilateral primary sensorimotor cortex, left V4, and left anterior intraparietal sulcus in response to a right hand complex ball-rotation task using a mirror. However, the mechanisms of MVF therapy remain controversial.

There are some limitations in fMRI studies of MVF, in that some patients had problems keeping their head still during the experimental task (Michielsen et al., 2011). However, the related technique of functional near-infrared spectroscopy (fNIRS) is considered to be a useful method for measuring neural activation under less constrained and more ecologically valid settings (Tuscan et al., 2013). Specifically, fNIRS does not require immobilization of subjects in constrained postures, and measurements can be done during motor tasks. Therefore, this technique is particularly advantageous for measuring neural responses when studying action execution (Balconi and Cortesi, 2016). Therefore in the present study, we investigated the influence of MVF on the hemodynamic response of the brain using fNIRS.

In recent years, there have been several bimanual MVF studies using passive movements by an experimenter (Fukumura et al., 2007) or electrical stimulation (ES; Yun et al., 2011; Lin et al., 2014; Nagapattinam et al., 2015; Lee et al., 2016). The effects of MVF combined with ES on motor and daily function have also been studied in patients with hemiparesis. The findings of these clinical trials suggest that there are numerous positive effects of MVF when combined with ES, including improved manual dexterity, grasping and transfer performance (Lin et al., 2014), Fugl–Meyer scores of hand and wrist coordination, as well as the power of hand extension (Yun et al., 2011; Kim et al., 2014), gait velocity, step length and slide length in gait ability (Ji et al., 2014), and muscle strength and balance (Lee et al., 2016). Although MVF with ES can be useful as a rehabilitation method, no neurophysiological studies of its underlying mechanisms have yet been reported. The present study used fNIRS and focused on the effects of MVF alone and also MVF along with ES to investigate hemodynamic changes in the brain.

## Materials and Methods

### Subjects

Eighteen neurologically healthy subjects participated in this study [seven males and eleven females; age, 24.9 ± 6.6 (mean ± standard deviation) years], and all subjects were right hand dominant according to the Chapman’s handedness test (Chapman and Chapman, 1987). This study was carried out in accordance with the Human Ethics Committee of Hokkaido University Hospital with written informed consent from all subjects. All subjects gave written informed consent in accordance with the Declaration of Helsinki. The protocol was approved by the Human Ethics Committee of Hokkaido University Hospital.

### Procedure

**Figure 1:**
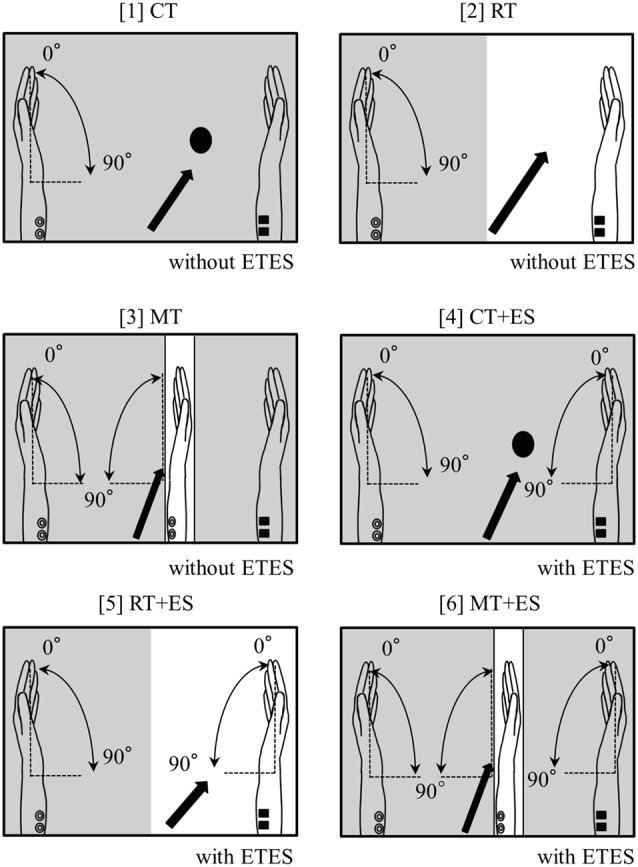
Schematic illustration of the experimental setup. The arrow in each task represents where the subjects were asked to look. The shaded portion indicates the areas that cannot be observed by subjects.

The subjects sat in a relaxed position in a reclining chair, and a custom-built mirror box was placed on a horizontal plate in front them. They were then asked to place both hands in a neutral position in the mirror box and perform six tasks, as illustrated in Figure 1, as follows. The subjects executed repetitive flexion movements of the left wrist in the box with a frequency of 0.5 Hz for 30 s in all tasks. They continued performing the wrist flexion and relaxation movements following the signal of a metronome in a personal computer:

1. *Circle Task (CT)*: while performing the task, the subjects visually fixated on a small round mark (1 cm diameter) on the box.
2. *Right-hand Task (RT)*: the subjects visually fixated only on their resting right hand.
3. *Mirror Task (MT)*: the subjects only watched a mirror reflection of the moving left hand superimposed on the resting right hand positioned behind the mirror.
4. *CT + ES Task (CT + ES)*: the right wrist was flexed by electromyogram-triggered ES (ETES) synchronously with muscle contractions in the left forearm.
5. *RT + ES Task (RT + ES)*: the subjects visually fixated only on their right hand being electrically moved.
6. *MT + ES Task (MT + ES)*: the subjects watched a mirror reflection with ETES as in MT.

The experimental design consisted of two stimulations and four rest periods. Each stimulation phase lasted 30 s, and each rest period lasted 20 s. As all of the tasks were performed three times in a random order, a total of nine cycles were performed, each lasting 140 s.

### ETES

The stimulation electrodes were placed 2–3 cm apart on the right flexor muscles of the forearm using rubber electrodes, and the triggered EMG was obtained from the left flexor carpi radialis using surface disposable Ag–AgCl electrodes (Vitrode F Disposable electrodes, NIHON KOHDEN, Tokyo, Japan). A grounded electrode was placed on the left olecranon. This method enabled moving the right hand by left hand movements and was developed by Futami et al. (2005). ETES is an approach in which ES is triggered by electromyographic activity. The frequency of ES is 20 Hz of 500 μs widths. The intensity of ES was set below each subject’s pain threshold but above their motor threshold before the start of the task, and we adjusted the sensitivity of the triggered EMG so that full joint movements could be elicited by ES.

### fNIRS Settings

**Figure 2:**
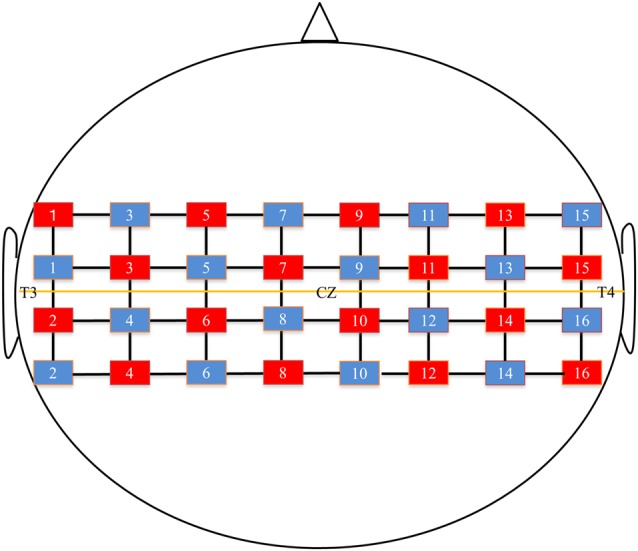
Near-infrared spectroscopy (NIRS) channels setting. The red square indicates the optical sources and the blue square indicates the detector. The midline shows T3-Cz-T4 line.

Hemodynamic activity was recorded using fNIRS (FOIRE 3000, Shimadzu, Co. Ltd., Kyoto, Japan), consisting of 16 optical sources and 16 detectors in a 4 × 8 probe array. The whole system consequently resulted in a total of 52 recording channels. The probe sets were bilaterally adjusted according to the international 10–20 system (Jasper, 1958) for electrode placement. Specifically, the channel for the 2nd tier between the 4th and 5th rows was located in the Cz position. In addition, the level between the 2nd and 3rd tiers was adjusted on a horizontal line of T3-Cz-T4 (Figure 2). The distance between the NIRS sources and detectors was set at 3 cm. Three different wavelengths (708, 805, 830 nm) with a pulse width of 250 μs were used to detect changes in cortical concentrations of oxygenated hemoglobin (oxy-Hb), deoxygenated hemoglobin (deoxy-Hb), and total hemoglobin (total-Hb).

To help obtain anatomical information, the location of optodes was marked with a 3D digitizer (FASTRAK; Polhemus, Colchester, VT, USA). This apparatus standardized the individual location of the NIRS channels to the skull shape of the subject. In addition, 3D T1-weighted MRI was performed for all subjects, and then using Fusion software (Shimadzu Co. Ltd.), the location of the NIRS channels marked with the 3D digitizer were superimposed on individual cortical surfaces. Then, the individual NIRS channels were classified into bilateral precentral gyrus (PrG), postcentral gyrus (PoG), supplementary motor area (SMA), supramarginal gyrus area (SMG), and angular gyrus (AG). We avoided the overlap of measurement positions because the spatial resolution of the fNIRS was 2–3 cm (McCormick et al., 1992); hence, this approach also excluded optodes on any cerebral sulcus from analysis. To standardize the amount of oxy-Hb change in all subjects, we confirmed whether the wavy line of oxy-Hb was in a stable condition before starting every task.

### Data Analysis

To explore fNIRS data, we used the oxy-Hb concentration changes, because previous studies have shown that oxy-Hb concentration is more sensitive to the change than deoxy-Hb and total-Hb in the local cerebral blood flow associated with brain neural activity (Murata et al., 2002; Fujiwara et al., 2004; Murkin and Arango, 2009). Changes in the oxy-Hb concentration were estimated based on a modified Beer–Lambert law (Seiyama et al., 2004). For baseline correction, the oxy-Hb data of each channel was normalized so that the average of values at 5 s before the task initiation was set to zero and we used a spline correction for data interpolation. A delayed reaction is an inherent characteristic in fNIRS recordings (Schroeter et al., 2004); hence, we calculated the value of the oxy-Hb concentration of every channel of NIRS during 25 s, from which the initial 5 s was excluded. The data obtained from each task were analyzed using 2 × 3 repeated measures analysis of variance (ANOVA) for each brain region with the factors “visual condition (VC; round mark, right hand, and mirror)” and “ES condition (ESC; with or without ES).” The Bonferroni correction for multiple comparisons was used for the *post hoc* *t-*test. Effects were considered significant at * p* < 0.05.

## Results

**Figure 3:**
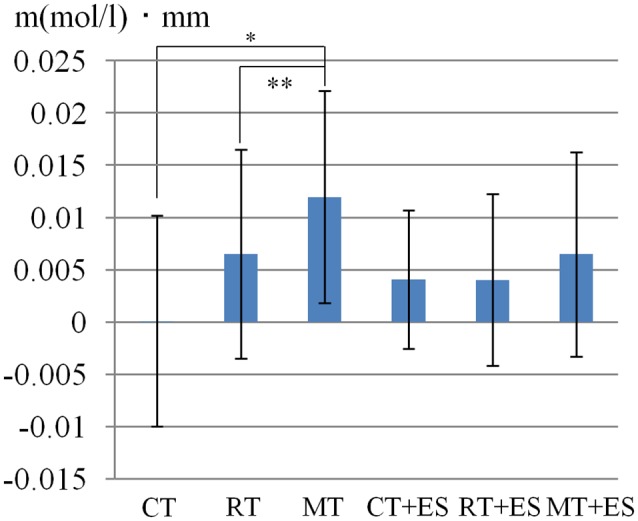
Oxygenated hemoglobin (Oxy-Hb) concentration in the left postcentral gyrus (PoG). *p < 0.05, **p < 0.01.

**Figure 4:**
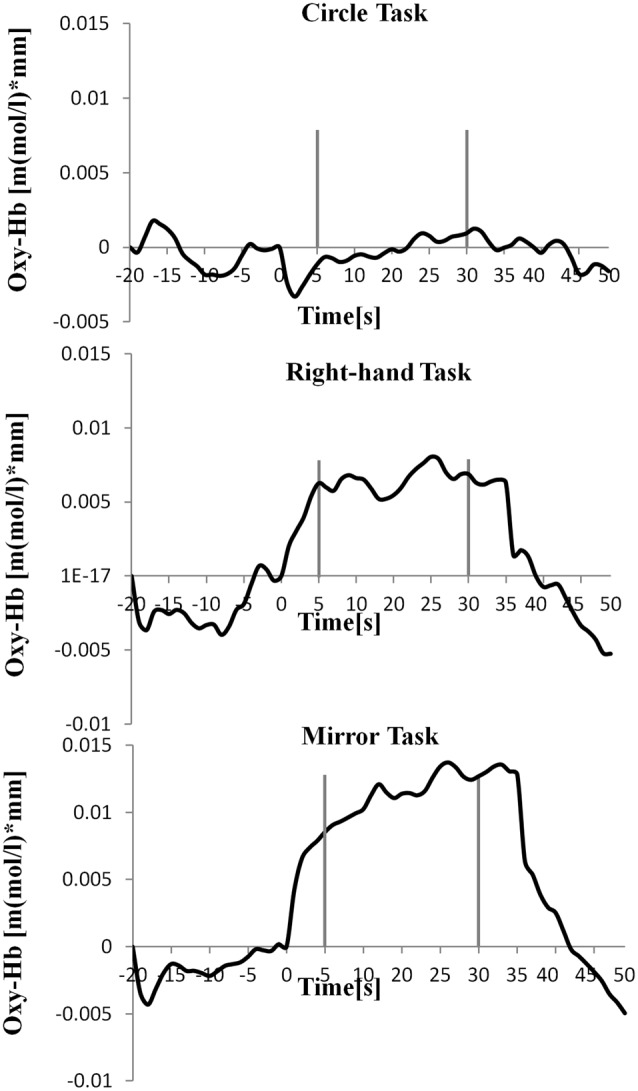
Time course of mean activation changes in oxy-Hb in response to Circle Task (CT), Right-hand Task (RT), and Mirror Task (MT) in left PoG that shows significant differences between groups.

**Figure 5:**
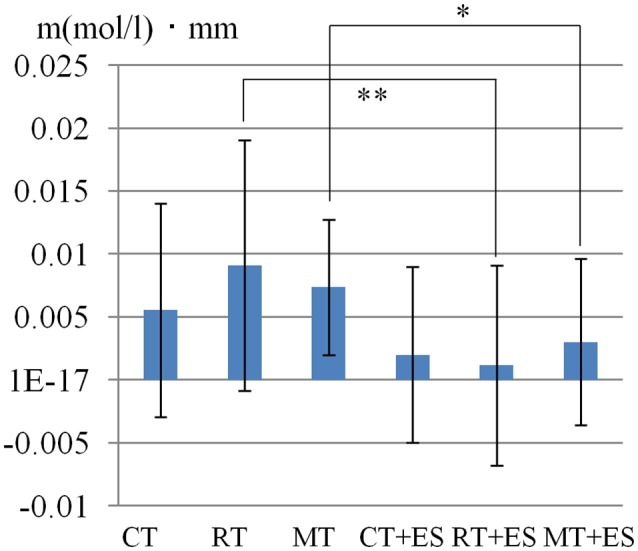
Oxy-Hb concentration in the right PoG. *p < 0.05, **p < 0.01.

**Figure 6:**
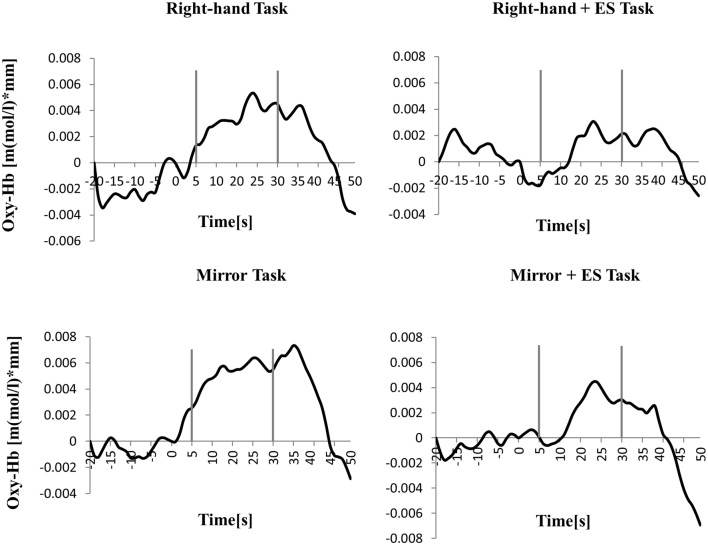
Time course of mean activation changes in oxy-Hb in response to RT, Right-hand + electrical stimulation (ES) Task, MT, and Mirror + ES Task in Right PoG that shows significant differences between groups.

**Figure 7:**
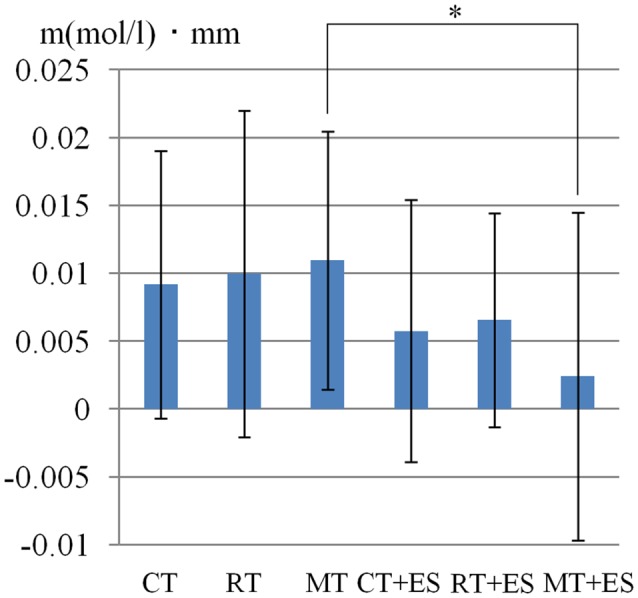
Oxy-Hb concentration in the right supplementary motor area (SMA). *p < 0.05.

**Figure 8:**
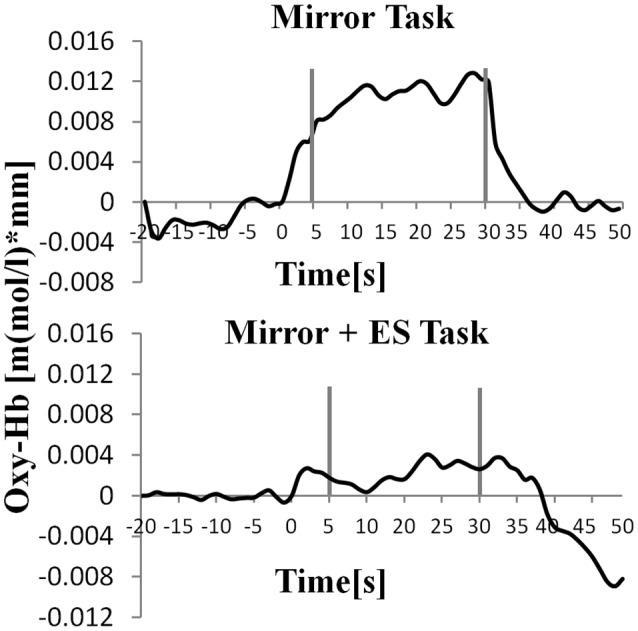
Time course of mean activation changes in oxy-Hb in response to MT and Mirror + ES Task in right SMA that shows significant differences between groups.

Table 1 shows the results of 2 × 3 repeated ANOVA tests. In addition, Table 2 shows the results of the Bonferroni correction for multiple comparisons. Regarding the left PoG, ANOVA revealed a significant main effect from VC (*F*_(2,34)_ = 8.23, *p* < 0.01) and VC × ESC interaction (*F*_(2,34)_ = 3.922, *p* < 0.05). A *post hoc* test showed that oxy-Hb concentration in MT was significantly higher than in both CT (*p* < 0.01) and RT (*p* < 0.05; Figures 3, 4). In the left SMA, ANOVA revealed a significant effect of ESC (*F*_(1,17)_ = 4.696, *p* < 0.05). Also, the oxy-Hb concentration without ES was significantly higher than that with ES (*p* < 0.05). In the right PoG and right PrG, there were significant main effects of ESC (right PoG: *F*_(1,17)_ = 8.02, *p* < 0.01, right PrG: *F*_(1,17)_ = 8.022, *p* < 0.05). The *post hoc* test showed that the oxy-Hb concentration without ES was significantly higher than that with ES (*p* < 0.01) and the oxy-Hb concentrations in both RT (*p* < 0.01) and MT (*p* < 0.05) were significantly higher than those in the ES tasks (RT + ES and MT + ES, respectively; Figures 5, 6). In the right SMA, ANOVA revealed a significant main effect of ESC (*F*_(1,17)_ = 6.365, *p* < 0.05), and the *post hoc* test showed that the oxy-Hb concentration without ES was significantly higher than that with ES (*p* < 0.05) and the oxy-Hb concentration in MT was significantly higher than that in the MT + ES case (*p* < 0.05; Figures 7, 8).

**Table 1:** Results of 2 × 3 repeated analysis of variance (ANOVA) conducted on VC, ESC and interaction effect for oxy-Hb changes of region of interests.

	Main effect (VC)		Main effect (ESC)		Interaction effect (VC × ESC)	
Region of interest	*F*	*p*-value	*F*	*p*-value	*F*	*p*-value
Left SMA	2.382	0.108	4.696	<0.05*	0.917	0.409
Left PrG	3.195	0.054	1.359	0.26	0.165	0.849
Left PoG	8.23	<0.01*	0.513	0.484	3.922	<0.05*
Left SMG	4.257	<0.05*	0.94	0.346	2.311	0.115
Left AG	4.489	<0.05*	0.448	0.513	0.477	0.625
Right SMA	0.288	0.687	6.365	<0.05*	1.289	0.289
Right PrG	0.355	0.703	8.022	<0.05*	0.119	0.889
Right PoG	1.056	0.356	8.02	<0.05*	1.524	0.232
Right SMG	0.181	0.836	2.78	0.114	0.015	0.985
Right AG	0.101	0.904	1.857	0.191	0.141	0.869

VC, visual condition; ESC, electrical stimulation condition; SMA, supplementary motor area; PrG, precentral gyrus; PoG, postcentral gyrus; SMG, supramarginal gyrus area; AG, angular gyrus; F, F-measure. *Indicate significant differences.

**Table 2:** Results of the Bonferroni correction for multiple comparisons.

Region of interest		Mean difference	Standard error	*p*-value
Left PoG	MT– CT	0.012	0.003	<0.01
	MT– RT	0.005	0.002	<0.05
Left SMA	ES− – ES+	0.005	0.002	<0.05
Right PrG	ES− – ES+	0.005	0.002	<0.01
Right PoG	ES− – ES+	0.005	0.002	<0.01
	RT– RT + ES	0.008	0.003	<0.01
	MT– MT + ES	0.004	0.002	<0.05
Right SMA	ES− – ES+	0.005	0.002	<0.05
	MT– MT + ES	0.009	0.003	<0.05

## Discussion

In the present study, we investigated the effect of traditional MVF alone and also along with ETES on the hemodynamic response of the cerebral cortex using fNIRS in normal young subjects. The results showed that the PoG ipsilateral to the moving hand was activated when the only stimulus was visual input from the mirror reflection of the moving hand rather than from the small mark and static right hand. In the ES tasks, the activation of left SMA ipsilateral to the moving hand, right PrG, right PoG, and right SMA contralateral to the moving hand decreased compared with that without ES. We also found that the cortical activation decreased compared with MT, especially in the responses of the right PoG and SMA resulting from MT + ES.

Several studies have demonstrated that MVF can facilitate the ipsilateral M1 excitation in observations of the mirror reflecting the moving hand (Garry et al., 2005; Debnath and Franz, 2016). However, the present study showed that the PrG ipsilateral to the moving hand in MT was not significantly activated more than in the CT and RT cases, but the PoG was significantly activated. As a possible explanation, Fritzsch et al. (2014) investigated the lateralization of the neural network attributed to hand movements under normal and mirrored (MIR) visual feedback conditions as well as during observation of a video clip of similar movements of a third person. The results showed that direct modulation was not elicited by the activation of M1 but by the primary somatosensory cortex in the MIR task. They suggested that MVF induced plasticity in M1 contralateral to the observed limb, but this should most probably be attributed to a training effect and not to an immediate and direct response to the mirror illusion itself. In contrast, the activity of the primary somatosensory cortex has the potential to be induced by attentional mechanisms or cross-modally by modulation of the visual image of a touched body part. Arya (2016) also reported in a review article that after receiving visuomotor messages, the primary somatosensory cortex on the lesioned side became immediately excited, while the premotor cortex activated later, following many MT sessions. In the present study, because the total time of the task was 30 s, our results demonstrated the immediate response to observation of the mirror reflecting the moving hand, which influenced the PoG ipsilateral moving hand.

However, we found that the oxy-Hb concentration of the left SMA, right PrG, PoG, and SMA in ESC tasks significantly decreased compared with those without ESC. Jang et al. (2014) have demonstrated that cortical activation of the sensorimotor cortex was decreased by neuromuscular ES. They suggested that the results were related to the motor learning effect. Toni et al. (1998) have also reported that a decrease in activation of sensory motor cortex following motor learning increased in normal subjects. In the current study, the oxy-Hb concentration of the right PoG was significantly decreased in right-hand observation tasks with ETES (RT + ES and MT + ES) compared with those without ETES (RT and MT) despite no significant change in the without-observation tasks (CT and CT + ES). Therefore, these results presumed that the combination of motor observation and synchronous movements with ES induced the motor learning effect, which decreased the cortical activation. Although the activation of the contralateral sensorimotor area decreased by combining the observation of the moving hand with ES, the cortical activations ipsilateral to the voluntarily moving hand were unaffected, presumably because of the short duration of the task. However, this study cannot comment in more detail regarding this topic based on the present evidence. Therefore, our results need to be evaluated regarding the short- and long-term effects of cortical hemodynamic activity in patients with hemiparesis to better understand the mechanisms underlying MVF with ETES.

On the other hand, the ES device used in the previous studies involves the use of an external switch (Kim et al., 2014; Lee et al., 2016), an electro mesh glove (Lin et al., 2014), and a preset electric stimulation cycle (Yun et al., 2011). In the ETES used in the present study, a general EMG-driven ES was used, wherein the input and output electrodes were attached to the opposite limb. This approach encourages the synchronization of the exercising limb reflected on the mirror and the electrically stimulated limb, thereby making it possible to derive the effects of MT + ETES. It is essential to compare and verify this approach with other stimulation devices in the future. Furthermore, it is possible to use an approach that combines MT and ETES to provide afferent input opposite to visual input. There exists a possibility that the mechanism of motor learning could be clarified by evaluating the temporal change of activity in the brain using such an approach. The limitations of this study include the small sample size and challenges associated with generalizing the results. In future research, it is necessary to adopt a larger sample size and compare the effect of mirror therapy with that of other interventions. In addition, research investigating the entire brain activation using fMRI during MT combined with ETES is warranted.

## Author Contributions

YI: experimental design, data acquisition, data analysis, data interpretation and manuscript preparation. HM: data acquisition of MRI. YM and TM: data analysis. KS and KI: data interpretation, manuscript preparation and revision.

## Conflict of Interest Statement

The authors declare that the research was conducted in the absence of any commercial or financial relationships that could be construed as a potential conflict of interest.

## Abbreviations

CT: circle task
ES: electrical stimulation
ESC: electrical stimulation condition
ETES: electromyogram-triggered electrical stimulation
MIR: mirrored
MT: mirrored tasks
VC: visual condition
RTs: right-hand tasks.
